# Ant-Plant Interaction in a Tropical Savanna: May the Network Structure Vary over Time and Influence on the Outcomes of Associations?

**DOI:** 10.1371/journal.pone.0105574

**Published:** 2014-08-20

**Authors:** Denise Lange, Kleber Del-Claro

**Affiliations:** Instituto de Biologia, Universidade Federal de Uberlândia, Uberlândia, Minas Gerais, Brazil; Universidade de São Paulo, Faculdade de Filosofia Ciências e Letras de Ribeirão Preto, Brazil

## Abstract

Plant-animal interactions occur in a community context of dynamic and complex ecological interactive networks. The understanding of who interacts with whom is a basic information, but the outcomes of interactions among associates are fundamental to draw valid conclusions about the functional structure of the network. Ecological networks studies in general gave little importance to know the true outcomes of interactions and how they may change over time. We evaluate the dynamic of an interaction network between ants and plants with extrafloral nectaries, by verifying the temporal variation in structure and outcomes of mutualism for the plant community (leaf herbivory). To reach this goal, we used two tools: bipartite network analysis and experimental manipulation. The networks exhibited the same general pattern as other mutualistic networks: nestedness, asymmetry and low specialization and this pattern was maintained over time, but with internal changes (species degree, connectance and ant abundance). These changes influenced the protection effectiveness of plants by ants, which varied over time. Our study shows that interaction networks between ants and plants are dynamic over time, and that these alterations affect the outcomes of mutualisms. In addition, our study proposes that the set of single systems that shape ecological networks can be manipulated for a greater understanding of the entire system.

## Introduction

Plant-animal interactions occur in a community context of dynamic and complex ecological interactive networks and the understanding of who interacts with whom is a basic information, but the outcomes of interactions among associates are fundamental to draw valid conclusions about the functional structure of the network [1]–[3].

Evolutionary ecologists are becoming increasingly interested in bipartite network analysis depicting interspecific interactions, as a tool for studies within an ecological context [4]–[6]. Bipartite network analysis includes several metrics, such as connectance, nestedness, cluster coefficients, web asymmetry, number of compartments, species degree, among others (see more details in [7]) and enables conclusions to be drawn on structure, stability and the robustness of interactions involving two groups of organisms [8]–[13] According to Hagen and co-workers [6], new data analytical tools such as network analysis, now form an essential ingredient in the study of complex systems.

In addition, experimental manipulations are widely used to explore the outcomes of interactions, mainly insect-plant interactions. The experimental methods vary according to the studied system. In a review [14], the authors argue that experimental manipulations also help to explore the structures that maintain preserved viable communities in an interactive way. In addition, some authors claim that long-term studies (e.g. intergenerational and/or temporal variation studies) and manipulative experiments within networks are fruitful avenues for future research on the mechanics of observed patterns in ecological networks [1], [15].

Although common and widespread in terrestrial ecosystems, extrafloral nectar-mediated ant-plant mutualisms have only recently been the focus of network analysis [16]–[29]. Ants are the main organisms linked to plant biotic defenses against herbivores and the most common resource that plants offer to attract ants is nectar produced by extrafloral nectaries (EFNs), a liquid substance rich in carbohydrates with dilute concentration of amino acids, lipids, phenols, alkaloids and volatile organic compounds [30]. The attracted ants might provide plants with effective protection against natural enemies [31], and carbohydrates of extrafloral nectar have been suggested to be a key resource for arboreal ants [32]. Ants feeding on extrafloral nectar increased their survivorship, colony growth and reproduction [33]. These mutualistic ant-plant interactions are highly facultative and vary in time and space, [34]–[35] mainly depending on the characteristics of the ant species [36]–[38], ant density [39]–[40], herbivore defensive strategies [37], as well as the plant species [41], and its phenology [29].

Although studies using experimental manipulation to explore the outcomes of this type of mutualistic interactions are relatively common in the literature (see [31], [42]), most of them have analyzed interactions between small subsets of ants and plants over a brief time interval [43]–[45]. The few studies that have directly investigated the effects of ants and EFN-bearing plant associations on communities, suggest that EFNs might influence species composition, abundance, and interactions on the community scale [41]–[42.], [46]–[47].

Here, we evaluate the dynamic of an interaction network between ants and plants with extrafloral nectaries, by verifying the temporal variation in structure and outcomes of mutualism for the plant community (leaf herbivory). To reach this goal, we used two tools: bipartite network analysis and experimental manipulation. Our main hypothesis is that ant-plant interactions are dynamic and exhibit temporal variation in their structure. This variation will influence the outcomes of mutualism, interfering within the effectiveness of ants as biotic control agents of plants against leaf herbivory. This type of study highlights the importance of considering the effects of variation in species composition, as well as their characteristics (i.e. natural history, morphology and behavior) in evolutionary ecology, which attempts to understand the patterns behind the topological features and functional structure of mutualistic networks.

## Materials and Methods

### Study site and plant characterization

Field study was conducted between September 2008 and April 2010 in a private natural Savanna reserve (Clube Caça e Pesca Itororó de Uberlândia/CCPIU - 48°17′ W; 18°58′ S) in Uberlândia, Minas Gerais State, south-eastern Brazil. The Biology Institute of Universidade Federal de Uberlândia has a memorandum of understanding with CCPIU, an agreement between Mr. Nilson Dias, head of CCPIU, and Dr. Kleber Del Claro, director of Biology Institute that enables ecological studies in the area. The vegetation is dominated by cerrado *strictu sensu*, consisting of trees 2–8 m in height, with an understory dominated by shrubs, grasses and scattered perennial herbs. As in other Cerrado areas, the climate in the region is rainy from October to April and dry from May to September (autumm-winter) [48].

Extrafloral nectaried plants are present in at least 25% of species and 31% of individuals in a Cerrado area [49] however, as verification of the interaction outcomes requires experimental manipulation, we chose a subset of plant species with extrafloral nectaries. The plant community chosen for this study corresponded to the most abundant EFN-tree species present in the study area, such as: *Caryocar brasiliense* (Cambess) (Caryocaraceae), *Lafoensia pacari* (A. St.-Hil.) (Lythraceae), *Ouratea spectabilis* (Mart.) Engl. (Ochnaceae), *O. hexasperma* (A. St.-Hil.) Baill (Ochnaceae), *Qualea grandiflora* (Mart.) (Vochysiaceae), *Q. multiflora* (Mart.) (Vochysiaceae), *Q. parviflora* (Mart.) (Vochysiaceae), *Stryphnodendron adstringens* (Mart.) Coville (Fabaceae) and *S. polyphyllum* (Mart.) (Fabaceae) (see [50]). The description of the location of EFNs in each species, as well as their morphology is found in [49], [51], respectively.

### Experimental manipulation and data collection for network construction

To identify which ant species visited the EFNs of each plant species and the effect of these ants on the leaf herbivory of the species, we conducted a long-term experimental manipulation. In August 2008, 30 similar individuals of each species (2–3 m tall, with a similar number of stems and phenology –without young leaves, buds, flowers and/or fruits) were selected. From each individual, we selected two similar stems (80 cm long) that were randomly designated as treatment or control. The treatment stems received a band of adhesive paper covering the whole circumference of the stem at its insertion with the trunk, and a layer of non-toxic ant repellent resin (Tanglefoot, Rapids, Michigan) was laid over strip. Ants already present were manually removed, as well as all structures that could act as bridges to provide access to the treatment stems. The control stems also received a band of adhesive paper with resin, however, it covered only half of the stem diameter, enabling ants to continue to climb the stem. In September 2008 and 2009, we tagged nine leaves on each stem of each individual. Three leaves were randomly chosen from each part of the stem: apex, middle and near the trunk insertion. The leaf area loss (leaf herbivory) of each leaf was examined in April (2009 and 2010), because after this month the most species lose its leaves resprouting in September or October. Therefore, in this study the data were collected into two periods: from September 2008 to April 2009 (corresponding to period 2009), and from September 2009 to April 2010 (corresponding to period 2010). Measurements of herbivory rates on leaves were assessed by placing the leaves on a transparent grid divided into millimeters. An index of herbivory from each leaf was calculated as the proportion of points in the grid falling within damaged and undamaged areas of the leaf blade [52]. The herbivory rate corresponded to the mean of the ratios of leaf damage assessed in the nine leaves. The proportions were evaluated in the field without leaf removal.

Fortnightly (from September to April), initially between 8:30 h and 10:30 h and subsequently between 14:00 h and 16:30 h, treatment stems were monitored for the integrity of the resin barrier and visiting ants from control stems were counted and identified. For the identification of unknown ant species, one individual of each species was placed in 70% alcohol. Ant species were identified at the Museu de Zoologia da Universidade de São Paulo (MZUSP) and voucher specimens were deposited at the Museu da Biodiversidade do Cerrado at the Universidade Federal de Uberlândia.

### Data analyses

To understand the network structure evaluated in this study, for each year, we carried out two incidence matrices (ants per plants), a qualitative matrix (considering the presence or absence of each ant species on each plant species) and other quantitative measurements (with frequency of interactions). We used the following metrics to check the properties of network interactions: connectance (sensu [53]), degree of species (*k*) and average degree (for plants and ants) (both sensu [54]), specialization index of the network (sensu [55]), nestedness index (sensu [56]), and web asymmetry (sensu [57]). We compared the frequency distribution of species per degree for ant and plant between years via a Kolmogorov-Smirnov two-sample test with the *D* statistic and we tested the differences in frequency distribution of species per degree between networks using XL-Stat Pro 7.52.

To measure the nestedness we used the *NODF* index (*Nestedness metric based on Overlap and Decreasing Fill*, see [56]), calculated by the software Aninhado 3.0 [58]. We estimated the significance of *NODF* using a Monte Carlo procedure with 1,000 randomizations, using a null model *Ce* (type II), in which the interaction probability between an ant and a plant is proportional to their total number of interactions. The *NODF* index is strongly recommended, due to its theoretical and statistical consistency [56].

We calculated the balance between the numbers of plant “*I*” and ant “*J*” species in each network using the following equation: *W =  (I−J)/(I+J)*, where “*W*” is the web asymmetry. Values equal zero for balanced webs; positive numbers indicate more plant species and negative values, more ant species; rescaled from −1 to 1 [57]. In addition, we calculated the network specializations using the *H_2_′* index (sensu [55]), which is a dimensional measure derived from Shannon's index and range from zero (extreme generalization) to one (extreme specialization) [55]. To calculate the network specialization index (*H_2_′*), we used the software R 2.13.2 (bipartite package) from the quantitative matrices. Finally, a bipartite graph of ant-plant interactions was constructed using the *Program for Analysis and Visualization of Large Networks* – Pajek 1.27 [59].

Since data from leaf loss area exhibited a normal distribution, they were analyzed using factorial ANOVA and Tukey post hoc comparisons. To compare herbivory between control (with ants) and treatment (excluding ants) stems in each plant species, we used the Student's *t*-test adjusted with the Bonferroni correction. Data for ant abundance were log(x+1) transformed and compared using a paired *t*-test. These statistical analyses were performed in Systat 12.0. All data are included within the manuscript.

## Results

### Ant diversity associated with plants

A total of 31 ant species from six subfamilies and 14 genera were associated with the studied EFN-bearing plants between 2009 and 2010 (Table 1). The most common genera were *Camponotus, Cephalotes* and *Pseudomyrmex*. Seven species [*Brachymyrmex* sp.1, *Camponotus crassus* Mayr, 1862, *Camponotus blandus* (Smith, F., 1858), *Cephalotes pusillus* (Klug, 1824), *Pseudomyrmex gracilis* (Fabricius, 1804), *Pseudomyrmex flavidulus* (Smith, F., 1858), *Ectatomma tuberculatum* (Olivier, 1792)] were recorded on all plant species, and two of these, *C. crassus* (present in more than 56% of plant individuals) and *C. pusillus* (in more than 45% of plant individuals), were ant species with a higher abundance and frequency (Table 1). The highest richness of associated ant species was observed on the plants: *S. adstringens*, *S. polyphyllum* and *Q. grandiflora*, which exhibited 61.3% of all linked ant species (*n* = 19 species); followed by *O. hexasperma* (58%, *n* = 18 species), *Q. parviflora* (54.8%, *n* = 17 species), *Q. multiflora* and *O. spectabilis* (51.6%, *n* = 16 species); *C. brasiliense* (45.1%, *n* = 14 species) and *L. pacari* (41.9%, *n* = 13 species).

**Table 1:** Ant species associated with plant species studied.

		Plant Species									
Code	Subfamilies/Ant Species	1	2	3	4	5	6	7	8	9	Total (*n* = 270)
	**Formicinae**										
1	*Brachymyrmex* sp.1	3	7	5	8	4	10	2	9	5	**53**
2	*Camponotus* sp.1	1	0	2	1	0	0	0	0	0	**4**
3	*C. crassus* Mayr, 1862	15	18	17	12	19	20	15	16	20	**152**
4	*C. blandus* (Smith, F., 1858)	5	8	5	10	4	2	9	6	6	**55**
5	*C. trapeziceps* Forel, 1908	1	2	1	3	3	5	2	2	0	**19**
6	*C. leydigi* Forel, 1886	1	1	0	3	0	0	0	1	0	**6**
7	*C. lespesii* Forel, 1886	1	0	0	0	0	1	0	0	0	**2**
8	*C. vittatus* Forel, 1904	0	0	0	0	2	1	0	0	0	**3**
	**Myrmicinae**										
9	*Crematogaster erecta* Mayr, 1866	1	2	2	2	1	0	3	5	0	**16**
10	*C. bruchi* Forel, 1912	1	1	2	1	3	1	1	0	0	**10**
11	*Pheidole* sp.1	1	1	1	0	0	1	1	0	1	**6**
12	*P.* sp.2	0	2	1	1	1	0	0	0	0	**5**
13	*Solenopsis* sp.1	0	2	3	1	0	0	3	2	1	**12**
14	*S.* sp.2	0	0	1	1	1	0	4	0	1	**8**
15	*Cephalotes* sp.1	1	0	0	0	0	0	0	0	0	**1**
16	*C. pusillus* (Klug, 1824)	10	17	16	13	17	11	15	17	6	**122**
17	*C. bruchi* (Forel, 1912)	0	1	0	0	0	0	0	0	1	**2**
18	*C. atratus* (Linnaeus, 1758)	1	0	0	0	0	0	0	0	0	**1**
19	*Nesomyrmex spininodis* (Mayr, 1887)	2	0	1	2	1	0	0	3	0	**9**
	**Dolichoderinae**										
20	*Azteca* sp.1	2	6	1	3	4	2	0	5	4	**27**
21	*Linepithema aztecoides* Wild, 2007	0	0	0	0	0	0	0	1	0	**1**
22	*Forelius brasiliensis* (Forel, 1908)	0	0	0	0	0	1	0	0	0	**1**
	**Pseudomyrmicinae**										
23	*Pseudomyrmex* sp.1	0	1	1	0	0	1	0	0	0	**3**
24	*P.* sp.2	0	0	0	0	0	0	1	0	0	**1**
25	*P. gracilis* (Fabricius, 1804)	4	9	13	5	3	9	7	10	3	**63**
26	*P. flavidulus* (Smith, F., 1858)	6	14	4	5	3	5	5	18	1	**61**
	**Ectatomminae**										
27	*Gnamptogenys semiferox* Brown, 1958	0	1	0	0	1	0	1	0	0	**3**
28	*Ectatomma tuberculatum* (Olivier, 1792)	9	4	7	5	2	6	1	1	1	**36**
29	*E. edentatum* Forel, 1912	0	0	0	0	0	2	0	0	0	**2**
30	*E. planidens* Borgmeier, 1939	0	0	0	1	0	0	1	0	0	**2**
	**Ponerinae**										
31	*Pachycondyla villosa* (Fabricius, 1804)	1	1	3	0	1	0	0	0	1	**7**

**Figure 1:**
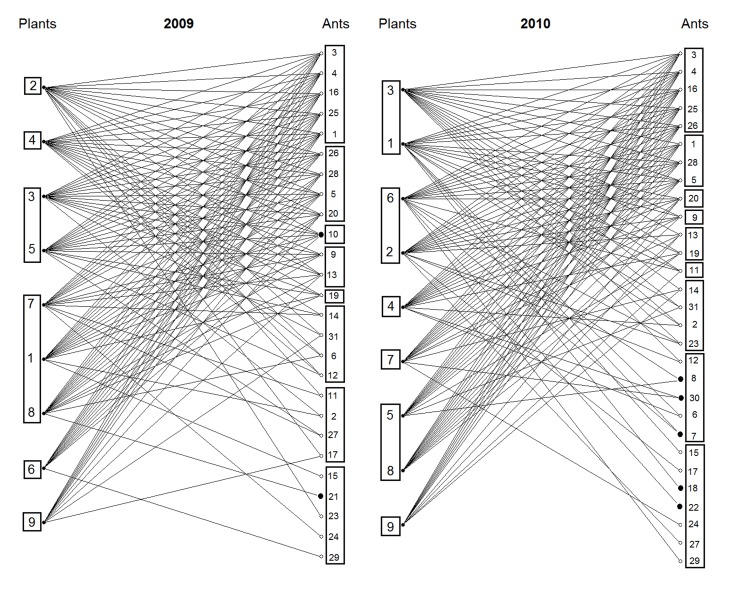
Ant-plant interaction networks in Brazilian Savanna studied in 2009 and 2010. Circles represent species, and links indicate extrafloral nectar feeding associations among ants and plants. Network 2009: 26 ant species and nine plant species; Network 2010: 29 ant species and nine plant species (see Table 1 for species identities). Bold circles in ant columns denote species that occurred in only one year. Rectangles denote species with the same degree.

Column numbers correspond to the total of individual plants (*n* = 30 trees per species) on which ant species were observed. Code numbers of plant species are as follows: (1) *Stryphnodendron adstringens*, (2) *S. polyphyllum*, (3) *Qualea grandiflora*, (4) *Ouratea hexasperma*, (5) *Q. parviflora*, (6) *Q. multiflora*, (7) *O. spectabilis*, (8) *Caryocar brasiliense* and (9) *Lafoensia pacari*. The code numbers of ant and plant species are the same as in Figure 1.

There was a change in the network interaction over time; in 2009, the network was composed of 26 ant species and two species: *Crematogaster bruchi* Forel, 1912 and *Linepithema aztecoides* Wild, 2007 were observed only in 2009. However, in 2010 the network was enlarged and comprise 29 ant species. Five species [*Camponotus lespesii* Forel, 1886, *Camponotus vittatus* Forel, 1904, *Cephalotes atratus* (Linnaeus, 1758), *Forelius brasiliensis* (Forel, 1908) and *Ectatomma planidens* Borgmeier, 1939] were recorded only in 2010 (Figure 1). Another five ant species were considered the most generalist, interacting with all plant species in both years: *C. crassus, C. blandus, C. pusillus, P. gracilis* and *P. flavidulus*. With almost equal importance and perhaps as generalist as the other species, *E. tuberculatum* and *Camponotus trapeziceps* Forel, 1908 were associated with eight out of nine plants in both years. These seven species maintained the same degree (number of links) over time and were also the most frequent according to their presence in the tagged individuals (*n* = 30) of each plant species (Figure 1). The ants *Crematogaster erecta* Mayr, 1866, *Nesomyrmex spininolis* (Mayr, 1887), *Solenopsis* sp.2, *Pachycondyla villosa* (Fabricius, 1804), *Pheidole* sp.2, *Pheidole* sp.1, *Camponotus* sp.1, *Gnamptogenys semiferox* Brown, 1958, *Cephalotes bruchi* (Forel, 1912) and *Pseudomyrmex* sp.1, also maintained their degree. The other 14 species demonstrated degree variation between 2009 and 2010. Some ants were considered as specialists, interacting with only one plant species: *Cephalotes* sp.1, *Pseudomyrmex* sp.2 and *Ectatomma edentatum* Forel, 1912 in both years; *L. aztecoides* and *Pseudomyrmex* sp.1 in 2009; and *Cep. bruchi, C. atratus, F. brasiliense* and *G. semiferox* in 2010. *Stryphnodendron polyphyllum* in 2009 and *Q. grandiflora* and *S. adstringens* in 2010 were the plant species that showed the greatest degree, with 17 links in each year. The plant with the lowest number of interactions was *L. pacari* with 10 links for each year.

### The structure and dynamic of the interaction network

The properties of the interaction network varied over time, but the pattern of nestedness and asymmetry was maintained. In 2010, there was a greater number of ant species interacting with plants and a greater number of interactions (Table 2). However, in 2009, 53.84% (*C*) of potential links were realized, whereas in 2010, this was reduced to 47.89%. Although the number of interactions varied between species and years (see Figure 1), the average degree was similar for plants (Network 2009 = 14±0.745; Network 2010 = 13.88±0.790, mean ± SE) and ant species (Network 2009 = 4.84±0.62; Network 2010 = 4.31±0.579). For ants, the degree varied between one and nine, but the more common degrees between species were 1, 2, 3, 8 e 9 in both years. For plants, the degree varied between 10 and 17, with a similar distribution between years. Following this tendency, the frequency distribution of degree did not vary between years either for ants (Kolmogorov-Smirnov, *D* = 0.111; *P*>0.05) or for plants (Kolmogorov-Smirnov, *D* = 0.059; *P*>0.05). Both webs showed asymmetry (2009 = 0.48; 2010 = 0.52), nestedness (2009 = 49.14; *P*<0.001; and 2010 = 45.11; *P*<0.001), and a low degree of specialization (0.104 and 0.128 for 2009 and 2010, respectively).

**Table 2:** The metrics of the interaction network for the association of ant-extrafloral-bearing trees.

Network metrics	Network 2009	Network 2010
Number of ant species	26	29
Number of associations	1195	1271
Degree of plant species (Average degree ± SE)	4.84±0.62	4.31±0.579
Degree of ant species (Average degree ± SE)	14±0.745	13.88±0.790
Network connectance	53.84%	47.89%
*H_2_'* index	0.104	0.128
Web asymmetry	0.48	0.52
Specialization asymmetry	−0.48	−0.52
Nestedness value (NODF)	49.14; *P*<0.001	45.11; *P*<0.001

### The outcomes of interaction

**Figure 2:**
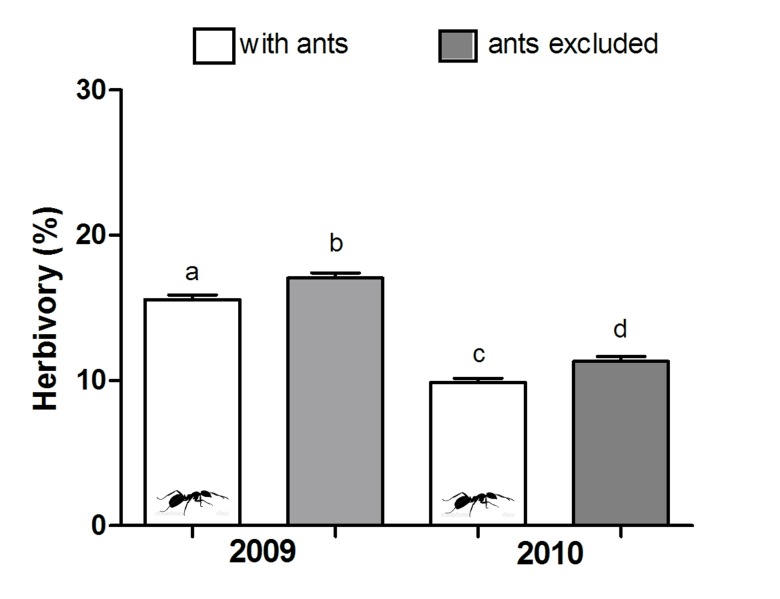
Comparison of herbivory between plants with or without ant exclusion. Different letters represent a statistical difference (*P*<0.05; ANOVA and Tukey post hoc comparison; Table S1). The horizontal line represents the mean, boxes represent standard error and whiskers, the standard deviation.

**Figure 3:**
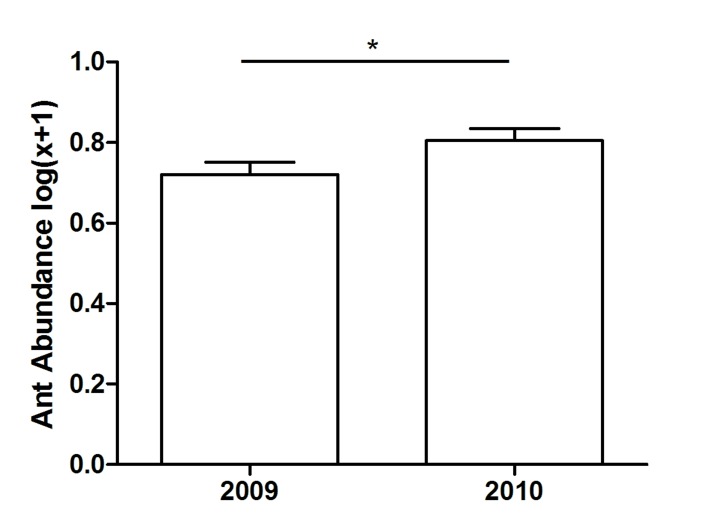
Ant abundance in stems with ants in the core of extrafloral nectaried trees. The asterisks represent statistical difference (paired *t*-test, *P*<0.05). The horizontal line represents the mean, boxes represent standard error and whiskers, the standard deviation.

**Figure 4:**
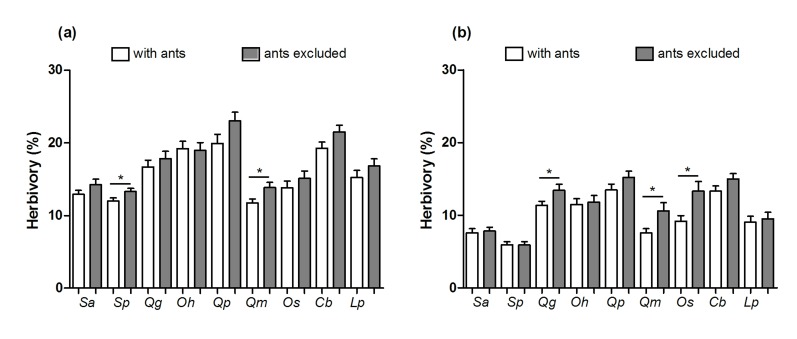
H**erbivory between stems with ants or ants excluded in the extrafloral nectaried trees.** Abbreviations: (Sa) *Stryphnodendron adstringens*, (Sp) *S. polyphyllum*, (Qg) *Qualea grandiflora*, (Oh) *Ouratea hexasperma*, (Qp) *Q. parviflora*, (Qm) *Q. multiflora*, (Os) *O. spectabilis*, (Cb) *Caryocar brasiliense* and (Lp) *Lafoensia pacari*; (a) 2009 and (b) 2010. The asterisks represent a statistical difference (*t*-test, *P*<0.05). The horizontal line represents the mean, boxes represent standard error and whiskers depict 95% intervals.

Leaf herbivory varied temporally and according to plant species and treatment (Table 3 and Table S1). Herbivory was higher in 2009 (Figure 2) and ant abundance on plants was higher in 2010 (*t* = −2.55; *P* = 0.01; Figure 3). In general, there was significantly greater herbivory in ant-excluded stems in both years (Figure 2). However, only four plant species (*Q. multiflora* and *S. polyphyllum* in 2009; *Q. multiflora*, *Q. grandiflora* and *O. spectabilis* in 2010) had a lower leaf area loss in stems visited by ants than ant-excluded stems (see Table S2). Independently of stem treatment, leaf area loss in both years was significantly higher in *Q. parviflora* and *C. brasiliense* than in other plant species (Figure 4 and Table S3).

**Table 3:** Results of ant-exclusion experiments.

Variables	df	Mean Square	*F*-ratio	*P*-value
Species	8	1056.642	53.636	0.000*
Stems	1	591.317	30.016	0.000*
Years	1	8472.147	430.056	0.000*
Species and Stems	8	24.820	1.260	0.261
Species and Years	8	60.391	3.066	0.002*
Stems and Years	1	0.000	0.000	0.997
Species, Stems and Years	8	13.696	0.695	0.696
Error	1016	19.700		

Factorial ANOVA comparing the herbivory (% leaf area loss) and variables: plant species, stem type (with and without ants), and year.

* statistical difference.

## Discussion

### The structure and dynamic of the interaction network

The networks studied here, exhibited the same general pattern as other mutualistic networks: nestedness, asymmetry and low specialization (see Table 2 and Figure 1), and this pattern was maintained over time. Our data and those of others [9], [20]–[21], [29], [42], showed that interaction networks between ants and plants with EFNs are shaped by a few generalist species and by multiple species that are linked to one or a few species. Specialization in this type of interaction is rare [42] however, as we observed, due to occasional ant or plant distribution (differential abundance, capacity of dispersion and colonization), species with low links or only one link are common, and might act, at least temporally, as specialists. Furthermore, seasonal variations might occur on the ant fauna associated with liquid resources (i.e. extrafloral nectar; honeydew) found on plants [41], [60], which can also explain the observed high amount of a few connected species [20]–[21], [29].

We also showed that not only the degrees of ant species changed over the time, but also those of plant species. A major number of ant species foraging on plants also means an increase in predation strategies against herbivores, what to the plant community means complementary effects (functional complementarity) of ant services (e.g. [60]). In plant-pollinator networks, from the consumer's viewpoint, differences in flowering phenology and/or nutritional variation in floral resources might explain a complementary role of different flower species [61]. Knowing that temporal and phenological variation also occurs in extrafloral nectar production [29] the same might be expected from studies of ant-plant systems.

In our data, the connectance decreased from 2009 to 2010; this was probably due to the effect of the absence in 2010 of one highly connected ant species observed in 2009 (*Crematogaster bruchi*) and the occurrence in 2010 of five different and less connected species in 2010 (Figure 1 and Table 1). Other studies in mutualistic networks [21], [62] also demonstrated that the incorporation of new species into the network did not interfere with the web structure, thus, new species interact with generalists and maintain the nested aspect of the network. Therefore, most generalist species in the network (hubs, those with a large number of interactions) become responsible for the maintenance of the network properties (see also [9], [11], [27]. This fact was demonstrated in this study by permanency of the species *C. crassus, C. blandus, C. pusillus, P. gracillis* on top of the network from year to year.

The existence of a few dominant species and many non-dominant species (subordinate and opportunistic species - [63]) in the community, contributes to the increase in the coexistence of these species in plants and, consequently, to the nestedness pattern of the network. Conversely, hierarchically superior species tend to be more abundant and frequent, as the model predicts the dominance hierarchy [47].

Although the default network has been maintained over time, about 50% of plant species and ants had a degree that was unchanged. This can be explained by the variation in the attractiveness of plant species as well as by the dynamics of the ant community. Several authors have observed variation in EFN-bearing plant attractiveness to ants and have related this variation to soil quality [26], plant biology and herbivory level [64], and abiotic factors [25].

Although variation in the degree of ant and plant species between the years studied was observed, the asymmetry, nestedness and specialization degree of the network maintained similar rates (Table 2). This result reinforces the idea that the general pattern of the network between ants and plants with EFNs does not vary over time, despite changes in the internal network (e.g. species degree and connectance) [29], [65]. The degree of specialization of the two networks studied was similar to that seen in other networks involving interactions between ants and plants with EFNs (see [18]). Despite the existence of interspecific competition among ants for the best resources (plants that produce nectar with higher quality), each ant visits such plants only for short periods of time, and does not necessarily nest on the plants, making interactions between ants and EFN-bearing plants non-specialized (a low *H_2_'*) [18].

### The outcomes of interaction

Most ant-plant experimental manipulative studies have historically been directed toward demonstrating ant benefits to plants and were carried out in systems exhibiting a unique producer (reviewed by [31]). Only recently has the viewpoint of ant fitness adequately been explored [33], [66], and the present study is the first to experimentally investigate the beneficial effect of ants on the ant-plant interaction network. Our results indicate that some general patterns viewed in singular systems are also present in a network subset. For example: a) the variation over time in the outcomes of benefits observed on the plant community (Figure 2) also occur when single species are analyzed (Figure 4; e.g. [34]–[35]); b) the assembly of ant species linked to plant species varied between years in a network (Figure 1) as in single ant-plant associations (e.g. [60]). We must consider that this depends on ant species features such as abundance (Figure 3), dispersion capacity, body size, aggressive behavior (e.g. [36], [41], [67]) and also on herbivore defensive strategies [37], [68].

Several authors have associated the outcome of mutualism between ants and plants with the behavior of the ant species involved [33], [36], [67]. As pointed out by other studies in the Cerrado biome [68]–[70], *Camponotus* species are the main defenders in these ant–plant interactions. Species of this genus are very agile and aggressive, feeding not only on EFNs, but also preying upon small arthropods [45]. Some ant species, such as those in the genus *Cephalotes*, despite being abundant in tropical systems, do not benefit the plants and are considered parasitic ant-plant systems [38], [68].

Differences in the use of arboreal sugars by dominant and non-dominant ants (discussed in the previous section) suggest that arboreal sugar composition might affect ant assemblage composition and thus the range of ecological functions performed by those ants [71]. The variation in the attractiveness of plant species for the ants together with the physical and chemical defenses of plants, might explain the observed variation in the percentage herbivory between species recorded over time.

As mentioned earlier, attractiveness is related to biotic and abiotic factors, which vary temporally. This variation was evident in both degrees of plant species and herbivory between years. According to Del-Claro and Oliveira [60], the temporal variation in biotic and abiotic conditions of the Cerrado environment can greatly influence the results of mutualistic interactions. In this context, we showed that ant presence had a strong positive effect on the herbivory of the plant community. In this facultative mutualistic interaction (sensu [72]), several ant species are involved in the relationship and the outcomes of the interaction can be related to several factors, including the behavior of associated ant species [38], as well as temporal variation [73].

## Conclusion

In this study, we showed that ant presence had a strong positive effect on the herbivory of the plant community during the two periods evaluated. However, this effect was smaller in 2009 than in 2010. Additionally, in 2009 we observed lesser ant abundance and degree of specialization (*H_2_'* index) compared to 2010. These results show the importance of greater abundance and specialization of ants in ant-plant protection system.

In conclusion, the internal changes in the ant-plant network influenced the variation of the outcomes of these interactions to the plant species. We also showed that ant-plant interactions can be manipulated as in single systems, as subsets of networks, which provide a more general and broader vision of the processes occurring in the entire community. We suggest that studies such as this one should be encouraged, because they can increase knowledge concerning the importance of spatial and temporal variation in ecology of interactions.

## Supporting Information

Table S1 **Tukey test comparing the leaf area loss between stems with and without ants for the years 2009 and 2010.**
(DOC)

Table S2 **Comparison of leaf area loss between stems with and without ants for all plant species separately in 2009 and 2010.**
(DOC)

Table S3 **Tukey test comparing the leaf area loss of plant species between stems with and without ants over 2009 and 2010.**
(DOC)
