# Supplementary material for: Ant-Plant Interaction in a Tropical Savanna: May the Network Structure Vary over Time and Influence on the Outcomes of Associations?
Source: PLoS One. 2014 Aug 20;9(8):e105574. doi: 10.1371/journal.pone.0105574 (PMC4139372; doi:10.1371/journal.pone.0105574)
Supplement: Table S1 — Tukey test comparing the leaf area loss between stems with and without ants for the years 2009 and 2010. (DOC) [file pone.0105574.s001.doc]

**Table S1** Tukey test comparing the leaf area loss between stems with and without ants for the years 2009 and 2010.

| **Comparisons** | **Difference** | ***p*-value** |
| --- | --- | --- |
| Control and treatment 2009 | -0.015 | 0.005 |
| Control 2009 and 2010 | 0.058 | 0.000 |
| Control 2009 and treatment 2010 | 0.043 | 0.000 |
| Treatment 2009 and control 2010 | 0.073 | 0.000 |
| Treatment 2009 and treatment 2010 | 0.058 | 0.000 |
| Control and treatment 2010 | -0.015 | 0.011 |
