# Supplementary material for: Ant-Plant Interaction in a Tropical Savanna: May the Network Structure Vary over Time and Influence on the Outcomes of Associations?
Source: PLoS One. 2014 Aug 20;9(8):e105574. doi: 10.1371/journal.pone.0105574 (PMC4139372; doi:10.1371/journal.pone.0105574)
Supplement: Table S2 — Comparison of leaf area loss between stems with and without ants for all plant species separately in 2009 and 2010. (DOC) [file pone.0105574.s002.doc]

**Table S2** Comparison of leaf area loss between stems with and without ants for all plant species separately in 2009 and 2010.

|  |  | Sa | Sp | Qg | Oh | Qp | Qm | Os | Cb | Lp |
| --- | --- | --- | --- | --- | --- | --- | --- | --- | --- | --- |
| **2009** | ***t*-test** | -1.395 | -2.128 | -0.821 | 0.175 | -1.796 | -5.310 | -0.966 | -1.805 | -1.194 |
|  | ***p*-value** | 0.169 | 0.038 | 0.415 | 0.862 | 0.067 | 0.000 | 0.338 | 0.076 | 0.237 |
| **2010** | ***t*-test** | -0.297 | 0.008 | -2.074 | -0.271 | -1.502 | -2.354 | -2.713 | -1.594 | -0.389 |
|  | ***p*-value** | 0.768 | 0.993 | 0.043 | 0.788 | 0.138 | 0.022 | 0.010 | 0.116 | 0.699 |

Abbreviations mean: (Sa) *Stryphnodendron adstringens,* (Sp) *S. polyphyllum,* (Qg) *Qualea grandiflora,* (Oh) *Ouratea hexasperma,* (Qp) *Q. parviflora,* (Qm) *Q. multiflora,* (Os) *O. spectabilis,* (Cb) *Caryocar brasiliense* and (Lp) *Lafoensia pacari.*
