# Supplementary material for: Ant-Plant Interaction in a Tropical Savanna: May the Network Structure Vary over Time and Influence on the Outcomes of Associations?
Source: PLoS One. 2014 Aug 20;9(8):e105574. doi: 10.1371/journal.pone.0105574 (PMC4139372; doi:10.1371/journal.pone.0105574)
Supplement: Table S3 — Tukey test comparing the leaf area loss of plant species between stems with and without ants over 2009 and 2010. (DOC) [file pone.0105574.s003.doc]

**Table S3 Tukey test comparing the leaf area loss of plant species between stems with and without ants over 2009 and 2010.**

| **2009** | Sa | Sp | Qg | Oh | Qp | Qm | Os | Cb | Lp |
| --- | --- | --- | --- | --- | --- | --- | --- | --- | --- |
| Sa | - | **0.997** | **0.046** | **0.000** | **0.000** | **0.989** | **0.998** | **0.000** | **0.664** |
| Sp | 0.998 | **-** | **0.003** | **0.000** | **0.000** | **1.000** | **0.852** | **0.000** | **0.184** |
| Qg | 0.124 | 0.014 | - | **0.472** | **0.156** | **0.001** | **0.303** | **0.427** | **0.931** |
| Oh | 0.008 | 0.000 | 0.993 | - | **1.000** | **0.000** | **0.000** | **1.000** | **0.019** |
| Qp | 0.000 | 0.000 | 0.002 | 0.049 | - | **0.000** | **0.000** | **1.000** | **0.002** |
| Qm | 1.000 | 1.000 | 0.053 | 0.002 | 0.000 | - | **0.750** | **0.000** | **0.117** |
| Os | 0.999 | 0.899 | 0.497 | 0.077 | 0.000 | 0.987 | - | **0.000** | **0.978** |
| Cb | 0.000 | 0.000 | 0.104 | 0.582 | 0.965 | 0.000 | 0.000 | - | **0.015** |
| Lp | 0.573 | 0.155 | 0.997 | 0.742 | 0.000 | 0.362 | 0.943 | 0.008 | - |
| **2010** | Sa | Sp | Qg | Oh | Qp | Qm | Os | Cb | Lp |
| Sa | - | **0.338** | **0.000** | **0.000** | **0.000** | **0.693** | **0.001** | **0.000** | **0.517** |
| Sp | 0.803 | - | **0.000** | **0.000** | **0.000** | **0.001** | **0.000** | **0.000** | **0.001** |
| Qg | 0.000 | 0.000 | - | **0.992** | **0.211** | **0.001** | **0.912** | **0.673** | **0.002** |
| Oh | 0.031 | 0.000 | 0.925 | - | **0.021** | **0.037** | **1.000** | **0.199** | **0.084** |
| Qp | 0.000 | 0.000 | 0.844 | 0.117 | - | **0.000** | **0.007** | **1.000** | **0.000** |
| Qm | 0.309 | 0.002 | 0.284 | 0.987 | 0.003 | - | **0.197** | **0.000** | **1.000** |
| Os | 0.001 | 0.000 | 1.000 | 0.969 | 0.857 | 0.464 | - | **0.087** | **0.336** |
| Cb | 0.000 | 0.000 | 0.933 | 0.198 | 1.000 | 0.007 | 0.936 | - | **0.000** |
| Lp | 0.884 | 0.062 | 0.029 | 0.651 | 0.000 | 0.993 | 0.083 | 0.000 | - |

Abbreviations mean: (Sa) *Stryphnodendron adstringens,* (Sp) *S. polyphyllum,* (Qg) *Qualea grandiflora,* (Oh) *Ouratea hexasperma,* (Qp) *Q. parviflora,* (Qm) *Q. multiflora,* (Os) *O. spectabilis,* (Cb) *Caryocar brasiliense* and (Lp) *Lafoensia pacari.*
